# Security governance and health inequity in contemporary crises: a critical discourse analysis of securitization, exception-making, and responsibility displacement

**DOI:** 10.1186/s12992-026-01226-8

**Published:** 2026-06-23

**Authors:** Amir Khorram-Manesh, Harald De Cauwer, Dennis G. Barten, Krzysztof Goniewicz

**Affiliations:** 1 Department of Surgery, Institute of Clinical Sciences, Sahlgrenska Academy, University of Gothenburg, Gothenburg, Sweden; 2 Center for Disaster Medicine, University of Gothenburg, Gothenburg, Sweden; 3 Gothenburg Emergency Medicine Research Group (GEMREG), Sahlgrenska University Hospital, Gothenburg, Sweden; 4 Faculty of Medicine and Health Sciences, University of Antwerp, Wilrijk, Belgium; 5 Department of Neurology, Sint-Dimpna Regional Hospital, Geel, Belgium; 6 Department of Emergency Medicine, VieCuri Medical Center, Venlo, the Netherlands; 7 Department of Security Studies, Polish Air Force University, Dęblin, Poland

**Keywords:** Geopolitics, Crisis governance, Critical discourse, Health inequity, Securitization, Sanctions, Peace and security, Humanitarian access

## Abstract

Contemporary crises increasingly draw health systems, humanitarian access, medical supply chains, and civilian protection into security-oriented governance. While the health consequences of war and economic coercion, and geopolitical rivalry are widely documented, less attention has been paid to the discursive processes through which such consequences are rendered legitimate, unavoidable, or politically peripheral. This paper addresses this gap by applying a critical geopolitics framework and critical discourse analysis to examine how geopolitical narratives may structure health-relevant governance during crises. Drawing on a purposive, illustrative corpus - (i) a primary corpus of institutional and policy discourse (state communications, United Nations and World Health Organization documents, sanctions guidance, and international non-governmental organizations’ reports) and (ii) a secondary corpus of scholarly literature, the study identifies three recurring discursive mechanisms: securitization, exception-making, and displacement of responsibility. Across five illustrative case contexts—US–China geopolitical rivalry, the Russia–Ukraine war, the Israel–Palestine/Gaza crisis, Iran-related sanctions and protest governance, and US–Venezuela sanctions governance—the analysis suggests how security narratives can condition humanitarian access, reshape the practical meaning of civilian protection, and diffuse accountability for health harms. Rather than arguing that discourse alone causes health outcomes, the paper treats discourse as one governance condition among others: it influences what becomes politically thinkable and institutionally actionable while interacting with military, economic, legal, and health-system factors. The findings advance critical health geopolitics and peace and security scholarship by showing how health inequities can function as a diagnostic lens through which norm erosion and the normalization of exceptional governance become empirically visible.

**Clinical trial number**

Not applicable.

**Supplementary Information:**

The online version contains supplementary material available at 10.1186/s12992-026-01226-8.

## Introduction

Contemporary crises increasingly draw health systems, humanitarian access, medical supply chains, and civilian protection into security-oriented frameworks [1, 2]. This does not mean that conflict, coercive economic measures, and geopolitical rivalry are newly securitized domains, rather, the relevant shift is that the health-relevant infrastructures and humanitarian practices are increasingly governed through vocabularies of threat, resilience, strategic vulnerability, and emergency necessity. These governance frameworks do not only structure military and diplomatic action; they also shape civilian protection norms, humanitarian space, and the operational conditions under which health systems function. In conflict- and sanctions-affected settings, health services are disrupted, medical supply chains are constrained, and population-level determinants of health deteriorate [1–3]. While these health consequences are widely documented, less attention has been paid to the discursive processes through which such consequences are rendered legitimate, unavoidable, or politically peripheral.

Much of the existing literature treats health inequities arising in geopolitical crises as downstream or “collateral” effects of state action [4, 5]. Such framing can obscure the underlying political and discursive processes through which policies with foreseeable health consequences are legitimized—constructed as necessary, proportionate, or inevitable rather than recognized as contestable policy choices [6, 7]. This omission is significant because international norms, including international humanitarian law (the Geneva Conventions), the World Health Organization’s constitutional commitment to the highest attainable standard of health and United Nations interpretations of the right to health, presume that civilian protection and equitable access are not optional, even in emergencies [8–11]. Yet security rationales repeatedly re-order governance priorities in ways that can make health harms simultaneously visible (as humanitarian “needs”) and politically insulated (as unavoidable by-products of rivalry, war, deterrence, or enforcement).

This paper argues that understanding health inequity in geopolitical crises requires moving beyond descriptive accounts of outcomes toward analysis of the discursive environments in which crisis governance operates. Geopolitical narratives do not merely describe emergencies; they structure institutional priorities, delimit policy options, and shape accountability. The argument is not that discourse alone determines health outcomes, but that discourse functions as a structuring condition for crisis governance—shaping what becomes politically thinkable and institutionally actionable when health is at stake [12, 13]. In this framing, “health inequity” is not only an outcome to be measured; it becomes an analytic site in which the erosion of civilian protection norms, the routinization of exceptional measures, and the diffusion of accountability can be observed empirically.

The analysis is situated across interconnected scales of public, population, and global health [14]. The COVID-19 pandemic illustrated how fragmented systems and hospital-centric responses can amplify vulnerability and undermine resilience [15, 16]. Antimicrobial resistance (AMR) similarly demonstrates how local practices generate transnational risks through travel and trade [17, 18]. Although COVID-19 and AMR are not empirical cases in this study, they help illustrate the broader health-security nexus: health-system challenges are often governed through preparedness, surveillance, supply-chain resilience, border management, economic continuity, and national-security vocabularies [15–24]. International Health Regulations (IHR) reflect attempts to manage these risks collectively, while also revealing tensions between sovereignty and cooperation [11].

Health security scholarship shows that security framing can be productive when it mobilizes resources, coordination, and political attention [19–24]. This paper therefore does not treat securitization as inherently harmful. The concern examined here is narrower: when security framing becomes dominant, equity, access, and accountability may become conditional, temporary, or administratively displaced. This is particularly relevant when health-relevant infrastructures—such as supply chains, corridors, hospitals, and payment channels—are treated as strategic vulnerabilities rather than shared public goods.

In this study, securitization refers to the discursive movement through which health-relevant domains are represented as threats or strategic vulnerabilities requiring urgent or exceptional governance [25]. Exception-making refers to the authorization of departures from ordinary protection norms through claims of necessity, proportionality, temporariness, or emergency [26]. Responsibility displacement refers to language that diffuses agency by presenting harms as indirect, technical, logistical, market-based, or politically contested [27]. The manuscript contributes to health security and critical geopolitics by examining how these mechanisms appear in authoritative institutional discourse—rather than media or public opinion narratives—and how they shape the governance conditions under which health inequities become normalized.

Historical precedents show that geopolitical contestation can constrain humanitarian access and weaken multilateral health cooperation. In the interwar period, major powers’ withdrawal from the League of Nations signaled the erosion of collective governance, with implications for associated technical bodies such as the League’s health work [28]. In the same era, humanitarian organizations including the ICRC faced severe operational constraints in relation to Nazi crimes against civilians, illustrating how “neutral” humanitarian action can be bounded by security politics and state control [29]. Against this backdrop, this paper builds on these historical insights to examine comparable discursive mechanisms—securitization, exception-making, and responsibility displacement—in contemporary institutional crisis governance (2019–2025) and contributes to critical health geopolitics by examining how political power operates through institutional discourse in health-relevant crisis governance [30, 31]. Applying critical discursive analysis (CDA), the study identifies three recurring discursive mechanisms, within the analyzed corpus, through which security frameworks stabilize governance arrangements with health consequences: securitization, exception-making, and displacement of responsibility.

To address the qualitative gap of discourse-focused work, the paper incorporates illustrative excerpts per case from the institutional corpus—short examples of wording that performs discursive work (e.g., recoding health as “national security,” bounding protection through “pauses,” or repositioning mortality as politically contested and therefore governable without accountability).

### Aims and research question

This study asks: *How do dominant geopolitical institutional narratives structure the governance conditions under which health inequities emerge*,* are stabilized*,* and normalized during security-framed crises?* The analysis focuses on: (1) securitization; (2) exception-making; and (3) displacement of responsibility.

## Materials and methods

Because the corpus includes different actor types, the analysis does not assume discursive equivalence across institutions. State communications, multilateral humanitarian documents, sanctions guidance, and non-governmental organizations (NGO)/human-rights reporting are treated as distinct genres of governance discourse. The analysis therefore compares how mechanisms operate differently across institutional genres rather than aggregating them into a single undifferentiated discourse. Widely syndicated wire reports, where retained, are used only to document contested mortality claims or policy-adjacent discourse and are not treated as equivalent to World Health Organization (WHO), United Nations (UN), or sanctions-authority documents.

### Study design

To operationalize the focus of critical geopolitics on discourse and power, this study employs critical discourse analysis (CDA) as its primary qualitative methodology. CDA examines how language functions as social practice—how it produces and reproduces authority, legitimacy, and the boundaries of what is politically thinkable and institutionally actionable [12]. Here, CDA is used to identify framings, metaphors, and justificatory logics through which crisis and security governance becomes intelligible, normalized, and publicly defensible within institutional texts [12, 13].

Consistent with CDA, the analysis does not treat texts as neutral descriptions of events. Instead, it approaches institutional documents as sites where competing narratives of security, sovereignty, responsibility, and humanitarian protection are articulated and contested, with implications for civilian protection and health-relevant governance. The methodological aim is therefore to show how discourse structures the field of permissible policy action, not to claim that discourse alone determines material outcomes.

### Case selection

The analysis draws on five illustrative case contexts: US–China rivalry [32–34], the Russia–Ukraine war [35–39], the Israel–Palestine/Gaza crisis [40–43], Iran-related sanctions and protest governance [44–47], and US–Venezuela sanctions governance [48–50]. Together, the cases provide variation across rivalry, war, protracted humanitarian crisis, and sanctions governance, while enabling cross-case identification of recurring discursive mechanisms. Iran is included not only as a sanctions case but also because protest governance and contested mortality constitute a high-visibility site where discourses of legitimacy, threat, and responsibility displacement become explicit in public and institutional communication. Cases were selected for their prominence in contemporary security governance and their capacity to illuminate recurring mechanisms, rather than to provide exhaustive geopolitical coverage.

### Data sources and analytic corpus

This study draws on a broad interdisciplinary literature to contextualize the analysis. However, only a defined subset of texts constituted the analytic corpus for CDA. The design distinguishes between: (i) a primary corpus of authoritative institutional discourse (coded), (ii) a secondary corpus of scholarly literature (contextual; not coded), and (iii) normative anchor texts used to establish baseline obligations regarding the right to health and emergency governance (not coded). A small number of widely syndicated wire reports were included only where they were reproduced, cited, or operationalized within policy-adjacent or institutional discourse to document contested mortality claims; they were not treated as a core media-analysis corpus. Recent U.S. presidential messaging related to Iranian protesters was retained only as contextual material to illustrate how protest governance can become geopoliticalized. It was not treated as equivalent to WHO, UN, Office of Foreign Assets Control (OFAC), sanctions-authority, or human-rights reporting, and it was not used as primary evidence for the cross-case CDA mechanisms.

(i) **Primary corpus (coded; 2019–2025).** The primary corpus consists of publicly available authoritative institutional and policy documents produced between 2019 and 2026 that frame or operationalize health-relevant governance under security-dominated crisis conditions. It includes:

1. Official foreign policy statements, press briefings, sanctions guidance, and related communications by state actors. State communications were selected purposively to capture authoritative, governance-relevant discourse. Inclusion criteria were: (i) issuance by a state body with direct crisis governance authority; (ii) explicit reference to humanitarian access, protection of healthcare, medical supply chains, sanctions exemptions/licensing, or related operational constraints; and (iii) publication during high-salience periods in each case between 2019 and 2025. Only official transcripts/releases from government sources were included; commentary and non-official media content were excluded.
2. Resolutions, meeting records, statements, response plans, and guidance issued by WHO and relevant UN bodies (including Security Council and humanitarian coordination documents); and.
3. Reports from major international nongovernmental organizations (INGOs) operating in conflict- or sanctions-affected settings selected for mandate relevance and recurrent citation within UN and WHO coordination ecosystems.

The primary corpus was assembled across the five case contexts listed above. Including Iran reflects the study’s focus on how external coercive policy (sanctions governance) intersects with internal crisis governance (protest repression and contested mortality) in securitized discursive environments [45–47]. The CDA was undertaken primarily by A.K-M. and K.G. All primary-corpus documents were first imported into NVivo to support systematic organization, coding, memo writing, and retrieval of coded passages (NVivo, Lumivero, Burlington, Massachusetts, USA). A.K-M. conducted the first cycle of open coding, identifying recurrent words, phrases, metaphors, framings, patterns of agency, and justificatory logics related to security, threat, sovereignty, humanitarian access, civilian protection, health inequity, and accountability. During this stage, analytic memos were written in NVivo to document coding decisions, emerging interpretations, and reflections on contested meanings. In the second cycle, related open codes were grouped into focused analytical categories aligned with the study framework: securitization, exception-making, responsibility displacement, and counter-framing/rights-based contestation. NVivo was then used to compare coded passages across case contexts and institutional actor types, including state communications, UN and WHO documents, sanctions guidance, and NGO/human-rights reporting. K.G. reviewed selected documents, code applications, and interpretive memos for conceptual consistency. Ambiguities in code application were discussed between A.K-M. and K.G., and the refined interpretive framework was reviewed by H.D.C. and D.G.B. for plausibility and disciplinary coherence. Because the study follows an interpretive CDA design rather than a quantitative content-analysis design, intercoder reliability statistics were not calculated. This is consistent with established interpretive CDA traditions, where credibility is ensured through reflexivity, audit trail, and co-author validation rather than statistical agreement. The coding framework is provided in Supplementary Table 1.

(ii) **Secondary corpus (not coded).** Peer-reviewed scholarly literature informed sensitizing concepts (securitization, exceptionalism, norm erosion) and supported interpretation and contextualization.
(iii) **Normative anchors (not coded).** WHO and UN normative instruments were used as baseline references for “non-optional” obligations and the legal–ethical context of emergency governance [8–11].

### Corpus transparency

The primary corpus comprised 20 documents; the complete list, with issuer, document type, year, and case context, is provided in Table 1. The Table serves as a corpus-construction transparency tool rather than a presentation of findings.

**Table 1:** The primary corpus documents

Case context	Institutional setting	Main actor / issuer	Document type	Document / identifier	Year	Corpus role	Main policy or governance relevance
Cross-case / global	Global health norms	WHO	Constitutional / legal text	Constitution of the World Health Organization	1948	Normative anchor, not coded	Establishes right-to-health baseline and health as a fundamental human right
Cross-case / global	Global health governance	WHO	Governance instrument	International Health Regulations (2005), 3rd edition	2016	Normative anchor, not coded	Establishes collective emergency-governance framework and sovereignty/cooperation tensions
Cross-case / rights	Human rights norms	UN CESCR	General comment	General Comment No. 14: The right to the highest attainable standard of health	2000	Normative anchor, not coded	Provides baseline obligations regarding access, equity, and minimum core duties
US–China rivalry	Geoeconomic and supply-chain governance	White House	Executive order	Executive Order 14,017: America’s Supply Chains	2021	Primary corpus, coded	Frames medical and pharmaceutical supply chains through resilience, security, and strategic dependency
US–China rivalry	Health technology governance	WHO	Initiative statement	COVID-19 Technology Access Pool / Solidarity Call to Action	2020	Primary corpus, coded	Provides counter-discourse of health technologies as public goods and shared resources
US–China rivalry	Regulatory and supply-chain governance	US FDA	Implementation report / portal	Executive Order 14,017 on America’s Supply Chains: FDA updates	2024	Primary corpus, coded	Operationalizes supply-chain resilience and medical-product security language
Russia–Ukraine war	Armed conflict and civilian protection	UN Security Council / UN Meetings Coverage	Meeting record / press release	SC/15,761: Security Council briefing on attack on Okhmatdyt children’s hospital	2024	Primary corpus, coded	Documents attacks on healthcare and frames civilian protection within security-governance debate
Russia–Ukraine war	Health emergency governance	WHO	Emergency appeal	Ukraine: WHO Health Emergency Appeal	2024	Primary corpus, coded	Describes health-system needs, access barriers, costs, and operational constraints
Russia–Ukraine war	Health emergency governance	WHO	Annual report	WHO’s response to health emergencies in Ukraine: annual report	2025	Primary corpus, coded	Documents protracted health emergency, resilience, recovery, and system strain
Israel–Palestine / Gaza	Humanitarian and health emergency governance	WHO EMRO / oPt	Operational response plan	WHO oPt Operational Response Plan, April–December 2024	2024	Primary corpus, coded	Frames health response through access, operational constraints, and service continuity
Israel–Palestine / Gaza	Security Council humanitarian governance	UN Security Council	Resolution	S/RES/2712: humanitarian pauses and corridors	2023	Primary corpus, coded	Authorizes temporary humanitarian pauses and corridors as bounded protection instruments
Israel–Palestine / Gaza	Security Council humanitarian governance	UN Meetings Coverage	Meeting coverage	SC/15,496: adoption coverage for Resolution 2712	2023	Primary corpus, coded	Provides institutional justification and debate around temporary humanitarian measures
Israel–Palestine / Gaza	Humanitarian coordination	UN OCHA	Flash appeal	Occupied Palestinian Territory Flash Appeal, April–December 2024	2024	Primary corpus, coded	Quantifies humanitarian need and describes access, displacement, and operational barriers
Israel–Palestine / Gaza	Security Council humanitarian governance	UN Security Council	Resolution	S/RES/2720: aid mechanism and acceleration of assistance	2023	Primary corpus, coded	Institutionalizes aid coordination and access mechanisms under security conditions
Iran sanctions and protest governance	Sanctions governance	US Treasury / OFAC	Sanctions guidance / program page	Iran Sanctions: OFAC sanctions programs and humanitarian trade guidance	2025	Primary corpus, coded	Frames humanitarian exemptions, licensing, compliance, and enforcement-risk conditions
Iran sanctions and protest governance	Human rights and mortality reporting	Iran Human Rights	Protest mortality report	One Year Protest Report: At least 551 killed	2023	Primary corpus, coded	Documents contested mortality and state violence during protest governance
Iran sanctions and protest governance	Crisis reporting / mortality contestation	Associated Press	Wire report	Report on renewed unrest, crackdown, and disputed death tolls	2026	Contextual source, not core coded corpus	Used only to contextualize contested mortality claims and attribution struggles
US–Venezuela sanctions governance	Sanctions governance	US Treasury / OFAC	Guidance / general licenses / FAQs	Venezuela-related sanctions and humanitarian guidance	2023	Primary corpus, coded	Operationalizes humanitarian authorization through licensing and compliance pathways
US–Venezuela sanctions governance	State legitimacy and sanctions framing	US Department of State	Program page / policy statement	Venezuela-related sanctions	n.d.	Primary corpus, coded	Frames sanctions through legitimacy, democracy, repression, and security narratives
US–Venezuela sanctions governance	Policy oversight	US Congressional Research Service	Issue brief	Venezuela: Overview of U.S. Sanctions Policy	2025	Primary corpus, coded	Summarizes policy evolution, sanctions rationales, and humanitarian implications

Anchor = normative baseline (not coded); Primary (coded) = in NVivo; Context = secondary literature/background (not coded). The final column describes the analytic rationale used during corpus construction and should be read as a guide to coding expectations, not as a finding. *Mechanisms*: Sec = securitisation; Exc = exception-making; Disp = displacement of responsibility. “Primary corpus, coded” refers to documents imported into NVivo and analysed using critical discourse analysis. “Normative anchor not coded” refers to legal or normative texts used to establish baseline obligations concerning health, equity, and emergency governance. “Contextual source, not core coded corpus” refers to material used only to contextualize contested mortality or political attribution and not treated as equivalent to institutional governance documents

### Coding and analytical process

**Fig. 1:**
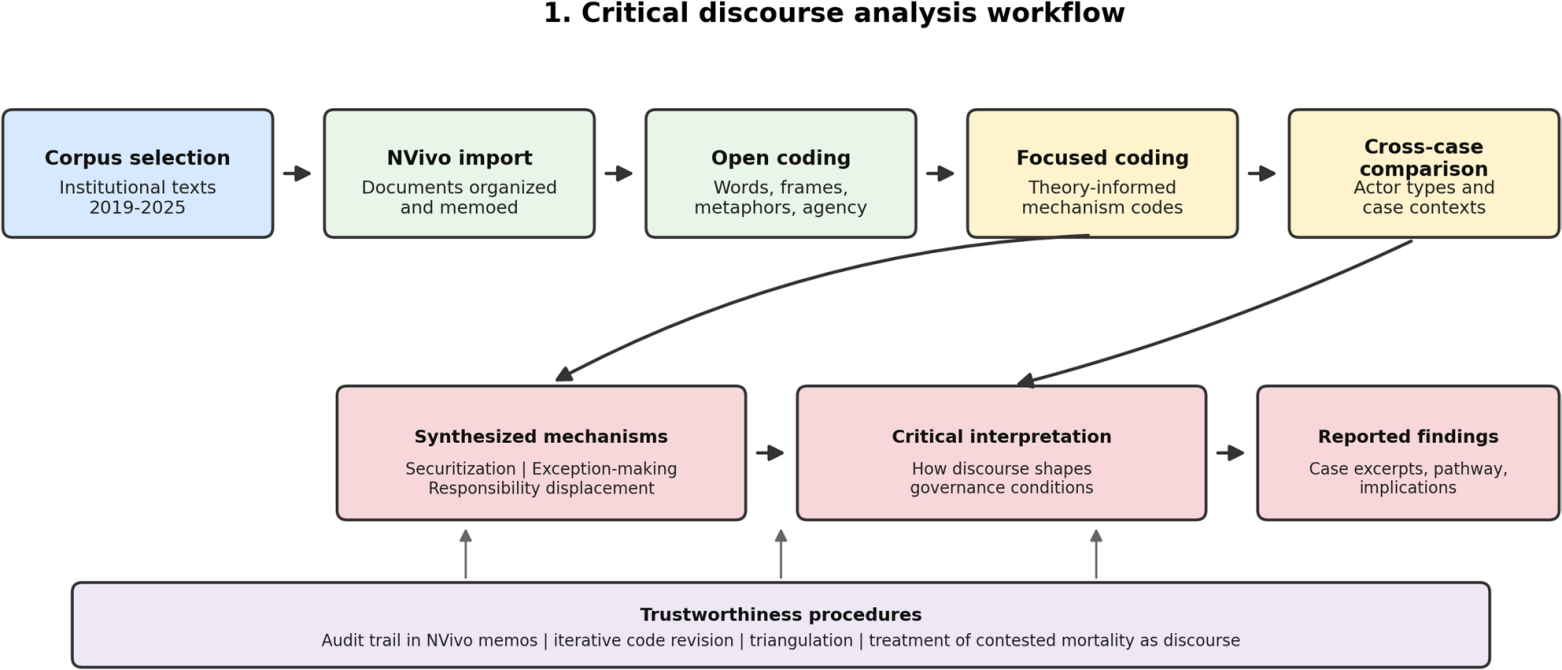
Critical discourse analysis workflow. The figure summarizes the progression from corpus construction and open coding to focused coding, cross-case comparison, synthesis of discursive mechanisms, and trustworthiness procedures. The workflow highlights the three primary mechanisms—securitization, exception-making, and responsibility displacement—alongside counter-framing and rights-based contestation

Keywords and codes were informed by critical geopolitics and CDA, focusing on security, threat, sovereignty, competition, legitimacy, and humanitarian concerns. All primary documents were analyzed using NVivo (Table 2; Fig. 1) [12, 30, 51].

**Table 2:** Analytical framework for coding discursive mechanisms in the institutional corpus

Discursive mechanism	Working definition used in coding	Textual indicators / coding prompts	Governance relevance	Illustrative case contexts
Securitization	The representation of health-relevant domains as threats, strategic vulnerabilities, or security assets requiring urgent, protective, or exceptional governance.	Does the text link health, medical goods, hospitals, humanitarian access, supply chains, or population movement to national security, threat, resilience, strategic competition, sovereignty, border control, or adversarial dependency?	Reorders health priorities by framing access, supply, infrastructure, or mobility as security concerns rather than primarily as rights, public goods, or equity obligations.	US–China supply-chain rivalry; Russia–Ukraine war; Gaza access governance; sanctions-related medical supply chains.
Exception-making	The authorization or normalization of departures from ordinary protection norms through claims of necessity, proportionality, temporariness, emergency, or operational feasibility.	Does the text use terms such as “temporary,” “necessary,” “proportionate,” “urgent,” “exceptional,” “pause,” “corridor,” “license,” “exemption,” “authorization,” or “emergency measure”? Does it frame protection as conditional or time-limited?	Makes constrained access, conditional protection, or special administrative arrangements appear necessary, temporary, or unavoidable, even when they become prolonged or repeated.	Gaza humanitarian pauses and corridors; Iran and Venezuela sanctions exemptions; emergency health appeals; conflict-related access restrictions.
Responsibility displacement	The diffusion, obscuring, or relocation of agency and accountability for health harms by presenting them as indirect, technical, logistical, market-based, environmental, or politically contested.	Does the text attribute harm to “operational constraints,” “the conflict environment,” “market conditions,” “compliance risks,” “logistical barriers,” “access challenges,” or disputed casualty figures? Are passive constructions used where agency could be specified?	Separates health harms from identifiable governance choices, making foreseeable consequences appear indirect, unavoidable, or beyond institutional responsibility.	Sanctions-related de-risking and payment barriers; Gaza access constraints; Ukraine health-system disruption; Iran contested mortality.
Counter-framing / rights-based contestation	Institutional language that resists or qualifies security-dominant framing by emphasizing rights, equity, civilian protection, humanitarian obligation, or health as a public good.	Does the text invoke the right to health, civilian protection, humanitarian principles, universal access, public goods, solidarity, non-discrimination, or minimum obligations? Is this language later narrowed by security or operational qualifiers?	Identifies moments where alternative governance logics remain visible, while also showing how they may be limited or reabsorbed into security-conditioned frameworks.	WHO C-TAP; WHO and OCHA humanitarian appeals; UN rights-based language; Security Council protection language.

Codes were applied interpretively rather than as mutually exclusive categories. A single passage could be coded under more than one mechanism when, for example, security framing was used to justify temporary constraints while also obscuring responsibility for downstream health harms. Counter-framing was coded to avoid forcing all evidence into the three main mechanisms and to identify moments where rights-based or equity-oriented language resisted securitized governance

### Analytical scope and generalization

The study is designed for analytic rather than representative generalization. It does not infer a direct causal chain from discourse to health outcomes. Instead, it examines discourse as one governance condition among others. The analysis uses “pathway” to refer to plausible institutional linkages through which framings can shape priorities, justify instruments, or delimit accountability. Material health outcomes also depend on military action, infrastructure, baseline health-system capacity, macroeconomic conditions, legal enforcement, and humanitarian access.

Analysis proceeded in three stages: (1) open coding; (2) focused coding aligned with securitization, exception-making, and responsibility displacement; and (3) critical interpretation linking discursive mechanisms to the governance conditions under which health constraints are rendered legitimate or unavoidable (Fig. 1).

### Trustworthiness and rigor

An audit trail was maintained through NVivo memos and iterative code revisions. Interpretations were triangulated against the secondary corpus and through systematic cross-case comparison to identify both shared mechanisms and context-specific variation [12, 13]. To reduce the risk of selective interpretation, illustrative excerpts were selected based on recurrence, institutional salience, and relevance to the coding categories. Given the politically contested nature of several contexts, particularly mortality reporting, the study treats the politics of naming, attribution, and counting as part of the discursive terrain rather than assuming stable empirical baselines [45, 47]. Additional procedures supporting analytic transparency and rigor are summarized in Supplementary Tables 2–4.

## Results

### Actor and context differentiation

The cases are not treated as functionally equivalent. State actors more often use threat, legitimacy, and strategic-resilience language; UN, WHO, and humanitarian texts more often use operational or technical language that documents harms while limiting explicit political attribution; sanctions authorities use permissive humanitarian language embedded in compliance architectures. The same mechanisms therefore operate through different institutional genres: supply-chain policy in US-China rivalry, war and health-emergency reporting in Ukraine, bounded humanitarian instruments in Gaza, sanctions/protest governance in Iran, and licensing/compliance discourse in Venezuela [32–50].

### Cross-cutting discursive mechanisms

Across cases, three recurring discursive mechanisms structured health-relevant crisis governance: securitization, exception-making, and displacement of responsibility. Here, securitization refers to the coupling of health-relevant domains with threat/strategic vocabulary; exception-making refers to the authorization of departures from ordinary protection norms through claims of necessity and temporariness; and responsibility displacement refers to causal framings that diffuse agency and accountability. These mechanisms interact: securitization establishes strategic priority, exception-making authorizes departures from ordinary norms, and responsibility displacement stabilizes accountability gaps. This configuration corresponds to what we conceptualize as a “permission-with-friction” governance architecture, in which access is formally enabled yet practically constrained through administrative, compliance, and operational conditions. Across the institutional corpus, this interaction repeatedly produced a recognizable governance pattern: health-relevant infrastructures (hospitals, corridors, supply chains, financial channels) were narrated as strategic risks; constraints were justified as necessary emergency measures; and health harms were documented yet framed as indirect, logistical, or politically disputed—thereby limiting accountability claims [52–56].

### Securitization of health and humanitarian domains

Across WHO communications, UN deliberations, state statements, and sanctions guidance, health-relevant domains—medical supply chains, humanitarian access, healthcare infrastructure—were consistently paired with security vocabulary (risk, threat, resilience, strategic vulnerability). Health is thus positioned as a strategic asset rather than primarily as a right or global public good within institutional crisis governance [32–50].

### Exception-making and normalization of constraint

Institutional discourse frequently invoked emergency, existential threat, or necessity to justify departures from humanitarian norms. Qualifiers such as “temporary,” “targeted,” “necessary,” and “proportionate” enacted exception-making by normalizing constraints affecting civilians, including movement restrictions, access barriers, and conditional protection of healthcare [50, 56].

### Displacement of responsibility

Health harms were often narrated as indirect effects, logistical challenges, market conditions, or consequences of the “conflict environment,” thereby attributing harm to abstract processes rather than to contestable governance decisions. This framing makes harms foreseeable yet politically insulated from accountability claims.

### Case studies

The following subsections illustrate how these mechanisms are instantiated in case-specific institutional vocabularies [39, 50, 52–55].

#### US–China geopolitical rivalry

Institutional discourse consistently framed health-relevant issues through the lens of strategic competition: supply-chain resilience, technological sovereignty, and risk reduction positioned interdependence as vulnerability [32–34]. Pharmaceuticals and medical technologies were treated as strategic dependencies requiring insulation from geopolitical risk, with health implications explicitly or implicitly marginalized as secondary to competitiveness and leadership [52–55]. This securitizing discourse is not merely rhetorical: it performs governance work by making “resilience” and “insulation” the default rationales for actions that reshape access and allocation across borders.

**Illustrative excerpts (institutional corpus)**:

1. From an FDA framing of supply chains under EO 14,017 [52]:

Keeping U.S. medical product and food supply chains secure, robust, and resilient is essential for the health and national security….

2. From a U.S. executive order on pharmaceutical stockpiling and production [53]:

Filling the SAPIR will… insulate the United States from… “foreign, sometimes adversary, nations” in… supply….

3. From a U.S. defense supply chain report [54]:

In times of crisis… demand… can… exacerbate economic and political differences between nations….

***CDA interpretation***: These excerpts enact securitization by explicitly coupling health commodities with national security and adversarial threat imaginaries. The “insulation” logic narrows what is institutionally actionable: policy attention shifts toward decoupling, redundancy, and strategic reserves, while equity-oriented frames (health as entitlement; health goods as global public goods) are displaced to the periphery of policy concern. In this discursive field, unequal access can be rendered not as governance failure but as a tolerable and even expected consequence of strategic resilience.

#### Russia–Ukraine war

Texts addressing the Russia–Ukraine war were structured around sovereignty and civilizational narratives [35–39]. Civilian infrastructure—including healthcare facilities—was discursively absorbed into security imaginaries [55, 56]. Even when international humanitarian law was acknowledged, the framing of existential conflict functioned to normalize prolonged disruption of healthcare delivery and narrowed space for accountability claims grounded in civilian protection. Health harms appeared as documented and tragic yet repeatedly positioned as an expected feature of war rather than a preventable governance failure.

**Illustrative excerpts** Primary institutional corpus, plus closely adjacent authoritative transcript reproductions and wire reports used to demonstrate discursive contestation:

1. From a widely reproduced text of the Russian leadership’s war framing [56]:

Russia sought to “demilitarize” Ukraine and pursue “denazification.”

2. From WHO reporting on attacks on healthcare [57, 58]:

WHO has documented over 2254 attacks on health care in Ukraine since the start of full-scale war….

3. From WHO emergency reporting language that converts attack patterns into governance imperatives [57, 58]:

Every delayed intervention worsens the situation and increases future costs.

##### CDA interpretation

The “demilitarization/denazification” framing performs exception-making by presenting extraordinary violence as necessary and morally compelled. The WHO excerpts, by contrast, document health harms through a technical evidentiary register. In WHO surveillance and incident-reporting systems (including SSA-style event reporting), attribution to specific perpetrators is often absent, with reporting framed around verified incidents and impacts rather than condemnation. This institutional neutrality can be operationally important, but it also widens the discursive gap between moralized war justifications and technical documentation; in turn, it can support responsibility displacement insofar as repeated attack patterns become legible as structural features of war rather than linked to contestable operational choices, enforcement failures, or civilian-protection obligations.

#### Israel–Palestine/Gaza crisis

Institutional discourse concerning Gaza was dominated by security narratives that structured whose vulnerability became visible and whose suffering was marginalized [40–43]. Constraints on movement and access were framed as security necessities. Humanitarian “pauses” and “corridors” enabled operational response while discursively bounding the scope of protection, sustaining a separation between “security policy” and “health outcomes” [59–63]. In this corpus, humanitarian need is extensively documented, yet the governance conditions producing need are frequently treated as “operating environment” constraints.

##### Illustrative excerpts(institutional corpus)

1. From an OCHA Flash Appeal statement that links success to “operating environment” shifts [59]:

This appeal requires critical changes… notably regarding access… and security….

2. From UN Security Council framing that bounds protection through temporary humanitarian measures [60–62]:

The Council called for “urgent and extended humanitarian pauses and corridors” in Gaza.

3. From OCHA crisis overview language documenting scale and thus stabilising “needs” discourse [59, 63]:

A “catastrophic” humanitarian situation with mass displacement and severe access constraints.

###### CDA interpretation

These excerpts illustrate how exception-making is enacted through the governance vocabulary of “pauses,” “corridors,” and “operating environment.” The effect is to make constraint administratively legible while narrowing the discursive horizon of what protection means (temporary, conditional, security-filtered), rather than rights-based guarantees. Importantly, this discursive effect does not depend on pauses reducing violence; attempted “pauses” may coexist with continued or even increased attack frequency, underscoring that the language of temporary relief can delimit the meaning of protection even when material risk remains high [64]. Responsibility displacement occurs when the “operating environment” becomes the implicit causal agent, enabling extensive documentation of health harms while backgrounding the specific policy architectures and enforcement choices that sustain access denial.

#### Iran-related sanctions and protest governance

Iran discourse combined moralized threat narratives with exception-making: sanctions were legitimized as security measures while humanitarian exemptions were affirmed in formal language [44, 65, 66]. Within this institutional framing, compliance risk and enforcement language foregrounded threats to financial and commercial actors, shaping how exemptions operated in practice and displacing responsibility for resultant health harms onto markets, logistics, and intermediaries.

Recent protest dynamics intensified the health stakes of securitized governance. The corpus shows how internal repression and external coercive policy can interact discursively: sanctions governance frames “exemptions” as sufficient safeguards, while protest governance disputes mortality and frames unrest as foreign-instigated threat—together producing a discursive environment in which both access barriers and death tolls can become governable without accountability. Longer-run impacts include disability burdens from trauma injuries, population mental health harms, deterrence from seeking care where surveillance is feared, and health workforce strain—effects that are readily narrated as “indirect” and thus politically insulated [45–47].

**Illustrative excerpts (institutional corpus and major-wire reporting treated as part of contemporary crisis discourse)**:

1. From U.S. sanctions/humanitarian guidance framing [44, 65, 66]:

Humanitarian exports (including “agricultural commodities, medicine, and medical devices”) are addressed as permissible channels.

2. From Iran Human Rights documentation of protest mortality (first-year “Woman, Life, Freedom”) [45]:

Iran Human Rights has verified the killing of 551 protesters including 68 children….

3. From contextual wire reporting on contested mortality claims [47]:

Iran Human Rights has verified the killing of 551 protesters including 68 children….

Activists alleged at least “6,221” killed; officials offered far lower counts and blamed “terrorists.”

***CDA interpretation***: The humanitarian-guidance excerpt performs exception-making by asserting the existence of exemptions—yet, in the broader sanctions’ corpus, exemptions are embedded in compliance-risk language that can discourage banks and suppliers (“de-risking”), thereby shifting the practical burden of access onto intermediaries. The protest mortality excerpts illustrate how casualty accounting becomes a discursive struggle: numbers, attribution, and legitimacy claims compete, and this contestation can itself function as responsibility displacement, because uncertainty about “true” mortality is used to defer accountability.

#### United States–Venezuela sanctions governance (moved into Results; expanded)

Institutional discourse around Venezuela framed the Maduro government through legitimacy/security narratives [48]. Humanitarian activity was governed as a licensed exception within a broader constraint regime: formal authorizations were enumerated in OFAC guidance, while practical access remained conditioned by compliance structures [49–50]. As in the Iran case, responsibility for downstream harms was often displaced by foregrounding formal permissibility while relocating failures to implementation layers (bank de-risking, transaction friction, administrative delay). In this case, delegitimating language (“illegitimate,” “usurpation,” “authoritarian”) works in tandem with exception-making (humanitarian licensing) to stabilize a coercive policy architecture while discursively insulating it from civilian health harms.

**Illustrative excerpts (institutional corpus)**:

1. From an OFAC humanitarian guidance framing [67]:

The guidance describes “support to the Venezuelan people” while operationalizing humanitarian activity through authorizations and compliance pathways.

2. From U.S. Treasury sanctions press release framing [67, 68]:

Treasury sanctioned officials tied to “repression” and an “illegitimate claim to power.”

3. From a U.S. State Department statement on legitimacy framing [69, 70]:

It referred to an “illegitimate presidential inauguration” and an attempt to “seize power.”

***CDA interpretation***: These excerpts show how delegitimating and securitization operate together: the target state is framed as an exceptional threat to democracy and regional order, authorizing coercive measures. Exception-making is enacted through humanitarian licensing and guidance that signals “permissibility,” while simultaneously embedding humanitarian action in a compliance architecture that can produce predictable friction and shortages. Responsibility displacement occurs when harms are attributed to domestic mismanagement or market behavior rather than to the sanction design and its compliance effects, thereby stabilizing the coercive regime as morally necessary while rendering civilian health consequences politically peripheral rather than governance relevant.

## Discussion

This study suggests how geopolitical discourse can operate as a structuring condition for crisis governance, shaping what becomes legitimate, necessary, or unavoidable in security-framed emergencies. Across five illustrative cases, securitization, exception-making, and displacement of responsibility interacted to narrow policy debate, normalize prolonged constraints on civilians, and diffuse accountability for foreseeable health consequences [71, 72]. Beyond documenting these mechanisms, the paper contributes by specifying how they translate into observable and recurring governance effects: the recoding of health systems and supply chains as security objects, the administrative routinization of “temporary” constraints, and the diffusion of agency through depoliticized or contested causal accounts. The qualitative excerpts presented in the Results section make this visible by showing how recurring institutional wordings (e.g., “national security,” “pauses and corridors,” “operational constraints,” and contested mortality claims) do not merely describe crises but help stabilize the boundaries of permissible action and responsibility. The aim is not to normatively adjudicate individual policy choices, but to make visible the discursive conditions under which certain health harms become politically acceptable or institutionally peripheral. A simplified governance pathway is discourse -> policy framing -> governance instrument -> operational condition -> health-relevant inequity indicator. For example, a “security threat” frame can support sanctions as necessary policy, which then produces licensing and exemption architectures; these can create bank de-risking, payment delays, supplier reluctance, and procurement friction; these operational conditions may then appear as medicine shortages, delayed access, or humanitarian delivery gaps. This pathway is not deterministic, but it clarifies how discourse can matter without replacing material explanations.

First, securitization recoded health-relevant infrastructures as strategic assets and vulnerabilities, rather than rights-bearing social systems. In US–China rivalry discourse, supply chains were explicitly framed as “national security,” and the language of “insulation” from “adversary” concentration legitimized policy trajectories that prioritize strategic resilience even when they risk fragmenting access [31–35]. This securitized framing matters for health equity because it shifts decision criteria from universal access and collective risk management toward control, redundancy, and strategic autonomy, thereby making uneven access and fragmented coordination appear as acceptable trade-offs rather than governance failures. In Russia–Ukraine, existential-war narratives and civilizational framings similarly absorb civilian infrastructures into the security imaginary, in ways that can weaken the discursive boundary between protected civilian space and military necessity [36–40]. In both contexts, securitization does not require explicit rejection of humanitarian norms; rather, it re-orders priorities so that protection of health systems becomes conditional on security logics rather than treated as a non-negotiable baseline.

Operationalizing health inequity as diagnostic: The diagnostic lens can be applied through observable indicators, including access disruptions, attacks on healthcare, supply-chain breakdowns, humanitarian payment friction, contested mortality, delayed rehabilitation, mental-health burdens, and workforce attrition. These indicators do not prove that discourse caused a given harm; rather, they help identify where governance language, policy instruments, and material inequities converge. For example, access disruption in Gaza may reveal how protection becomes bounded by “pauses,” “corridors,” and “operating environment” language; payment friction in Iran and Venezuela may reveal how humanitarian exemptions operate as permission-with-friction; contested mortality in Iran and Gaza may reveal how counting and attribution become part of responsibility displacement.

Second, exception-making stabilized prolonged constraint by narrating departures from ordinary protection norms as temporary, necessary, or operationally conditioned. In Gaza, the discourse of “pauses” and “corridors,” and the repeated emphasis on “operating environment” requirements, constituted a governance language that enables humanitarian action while bounding the scope of protection through security-conditioned access [59–63]. This boundedness is analytically important: it produces a narrower protection horizon in which relief is framed as an intermittent, conditional exception rather than a rights-based obligation, and where the practical meaning of “protection” is continually renegotiated through security criteria.

A related discursive move concerns the use of security labels—especially “terrorism” designations—to reorganize the moral and legal terrain of protection. Because definitions of terrorism are contested and can be strategically mobilized, labeling practices may narrow the civilian-protection horizon by shifting populations, spaces, or infrastructures into categories governed primarily through security imperatives [73, 74]. This politicization can function as exception-making by legitimizing expanded coercive measures (surveillance, movement restriction, access denial) as necessary and time-insensitive responses to “security threats,” even where such measures generate foreseeable health harms. It can also reinforce responsibility displacement by framing downstream civilian suffering as an unavoidable by-product of counterterrorism governance rather than as a contestable outcome of policy design, targeting choices, or enforcement architectures [75, 76].

Actor-specific policy implications: For WHO, UN, and humanitarian organizations, reporting could distinguish operational constraints from policy-imposed constraints where attribution is feasible and avoid passive constructions that erase agency. For Security Council members and states, bounded instruments such as pauses and corridors should be assessed not only for immediate feasibility but also for whether they normalize conditional protection. For sanctions authorities, humanitarian access should be designed as a safe and monitored governance pathway rather than a narrow exception, including clearer safe-harbor provisions, reduced licensing ambiguity, and monitoring of de-risking effects. For global health researchers, CDA should be triangulated with multilingual corpora, interviews, and longitudinal indicators of access, disability, mental health, workforce strain, and supply-chain disruption.

In sanctions contexts, humanitarian exemptions are repeatedly affirmed in formal language, yet their practical fragility is produced discursively through compliance-risk and enforcement rationales that recode humanitarian channels as governance risks to be managed [44, 50, 77, 78]. Here, exception-making works through a distinctive “permission-with-friction” architecture: humanitarian access is articulated as permissible in principle, while the surrounding compliance vocabulary (risk, liability, enforcement exposure) encourages overcompliance and de-risking, shifting the burden of access onto intermediaries and thereby normalizing constraint as an implementation problem rather than a predictable outcome of policy design.

Third, displacement of responsibility diffused accountability by narrating harms as indirect effects, logistical realities, or politically disputed facts. This mechanism was visible when harms were attributed to “market conditions,” “the conflict environment,” or “operating constraints,” rather than to contestable policy architectures. Displacement has a clear governance effect: it de-links injury and deprivation from decision points, narrowing the space for accountability claims and foreclosing alternative policy pathways. The Iran case illustrates this dynamic in an intensified form: protest mortality itself becomes contested and governable through competing numbers, attribution struggles, and information controls, which can function as accountability shields [45, 47, 77, 78]. Longer-run harms—disability from injuries, mental health burdens, fear-driven care avoidance under surveillance, and health workforce strain—are especially vulnerable to being rendered “indirect,” and thus politically insulated, because they are temporally lagged, harder to quantify, and easier to narrate as diffuse social consequences rather than governance outcomes. Importantly, contested mortality is not only a methodological challenge but an empirical feature of crisis governance itself, aligned with CDA: struggles over naming, attribution, and counting become part of the crisis governance environment in which responsibility is negotiated or displaced [79].

A further implication across cases is the presence of discursive contestation rather than total discursive closure [68]. Rights-based and humanitarian framings do appear—especially in public health and humanitarian reporting and in some institutional condemnations of attacks on healthcare—yet these counter-frames are frequently narrowed through operational or security qualifiers, which can reconstitute protections as conditional and partial [80, 81]. This tension is not incidental; it illustrates how norm assertion and norm erosion can coexist within the same governance field, producing repeated cycles in which obligations are recognized in principle but limited in practice through bounded instruments, administrative conditions, or compliance architectures [57–63, 82–86].

Relatedly, institutional “technical neutrality” can function ambivalently: it may protect institutional access and mandate in contested geopolitical environments, but it can also contribute to responsibility displacement by narrowing the institutional vocabulary available for naming political drivers of inequity, thereby limiting the capacity of health governance discourse to contest upstream determinants.

Dehumanization may function as a psychosocial condition that makes securitized and exceptionalist governance frames more politically sustainable in protracted crises. Research on reciprocal and meta-dehumanization shows links with outgroup hostility and support for aggressive policies, while public health studies connect such dynamics to trauma, restricted mobility, damaged infrastructure, and interrupted care. Dehumanization does not replace the mechanisms analyzed here, but it can interact with securitization and exception-making by narrowing the practical meaning of civilian protection in health governance [87–89].

Seen in this light, health inequity functions not only as an outcome of crisis governance but as a diagnostic signal of deeper transformations in security-oriented governance. Across the cases examined, disrupted access, constrained protection, and contested mortality become visible where securitization, exception-making, and responsibility displacement converge. Health impacts are therefore empirically revealing not because they are uniquely severe, but because they often show how exceptional security logics displace commitments to civilian protection and the right to health before such shifts are fully acknowledged in legal or policy terms [90].

### Implications for global health and crisis governance

Recognizing this diagnostic role has implications for global health, humanitarian governance, and peace and security scholarship alike, suggesting that attention to health outcomes can provide critical insight into when crisis governance moves from protection to managed constraint. These findings underscore the limits of purely technical or operational approaches to crisis preparedness. In politicized emergency environments, governance is shaped as much by narrative and legitimacy as by capacity or resources [79, 82, 88]. Health outcomes are therefore inseparable from the security discourses that structure decision-making. Where securitization dominates, equity and civilian protection are more easily subordinated to strategic concerns, undermining international commitments to the right to health and to cross-border cooperation in emergencies [8–11, 15].

These implications can be made actionable without requiring deterministic claims about discourse. For multilateral and humanitarian institutions, greater attention to language and agency can reduce responsibility displacement—for example, by distinguishing “operational constraints” from policy-imposed constraints where attribution is feasible, and by avoiding passive constructions that erase decision-makers when documenting preventable harms. In sanctions governance, policy actors can treat humanitarian access as a designed governance system rather than a formal exception by clarifying safe-harbor pathways, reducing ambiguity that drives overcompliance, and monitoring access interruptions as governance-relevant impacts rather than unintended side effects [44, 50, 71]. Across conflict contexts, the routine use of bounded protection instruments (such as “pauses” and conditional corridors) should be evaluated not only for immediate feasibility but for the ways these framings may normalize prolonged deprivation and weaken expectations of civilian protection [59–63]. More broadly, the findings point to the need for crisis governance that explicitly recognizes the political determinants of health rather than relegating them to background conditions.

Peace, stability, and clear dialogue are also public health issues [91]. When crises are framed mainly through security language, communication often becomes polarized (“threat,” “enemy,” “dictator”) and responsibilities are blurred [83–89]. This can reduce cooperation, delay access, and make civilian suffering feel structurally “unavoidable.” By contrast, clear and understandable dialogue—using precise terms, transparent explanations of decisions, and consistent rights-based language—can support de-escalation, improve coordination, and protect humanitarian space. In practice, sustained communication and credible messaging should be treated as part of crisis health-relevant governance infrastructure because they shape whether healthcare can be protected, aid can reach civilians, and accountability remains possible.

### Contributions and limitations

The paper advances critical health geopolitics by demonstrating how health inequity can be mobilized as an analytic and empirical surface on which norm erosion and exceptional governance become observable [92]. It contributes by showing (i) how recurring institutional wordings and justificatory qualifiers shape the operational and practical meaning of protection and access, (ii) how sanctions “exemptions” can operate as discursive-technical governance systems that normalize friction, and (iii) how contested mortality and attribution function as part of governance rather than merely as uncertain background data. Limitations include the interpretive nature of CDA [12], the absence of modelling of non-discursive determinants (baseline capacity, macroeconomics), and uneven verifiability of mortality and health harm in politically closed contexts where casualty reporting is itself contested [45, 47].

A further limitation is linguistic: the analytic corpus consisted of English-language documents or documents available in official English translation. Relevant Russian, Ukrainian, Spanish, Hebrew, Arabic, Persian, and other language sources may contain different rhetorical forms, justificatory logics, or counter-discourses. The purposive corpus supports mechanism-building and interpretive comparison but does not permit claims about the prevalence of these mechanisms across all crisis discourse. Finally, because the corpus includes heterogeneous actor genres, the analysis must distinguish state/security discourse, multilateral humanitarian discourse, sanctions-compliance discourse, and NGO/human-rights reporting rather than treating them as a single voice.

A further constraint is the limited ability of international actors to deploy independent observers in many conflict and repression settings, due to access denial, security risks, and restrictions on monitoring mandates. This absence of on-the-ground verification can amplify contested narratives, complicate attribution, and enable responsibility displacement by making harm harder to document consistently and independently [82, 93]. Another limitation is that the excerpts presented are illustrative rather than exhaustive; while selection was guided by recurrence, issuer authority, and representativeness of mechanisms across the corpus, other selections and readings remain possible. Future research could triangulate CDA with interviews, ethnography, and quantitative health indicators, including longitudinal assessment of disability, mental health outcomes, and health workforce impacts in protracted crises.

In the Iran case specifically, future work could operationalize longer-run impacts through measurable and governance-relevant proxies (e.g., rehabilitation demand, trauma and mental health service utilization, workforce attrition, internet shutdown, and delayed-care indicators), triangulated with humanitarian access documentation and governance timelines [45, 47, 73, 79].

## Conclusion

Health inequities in emergencies are shaped not only by pathogens, violence, and scarcity but also by the narratives through which crises are governed. Across five illustrative case contexts, securitization, exception-making, and displacement of responsibility appeared in institutional and policy discourse in ways that can condition healthcare protection, humanitarian access, and accountability, thereby rendering foreseeable health harms legitimate, unavoidable, or politically peripheral. Recognizing these mechanisms is relevant to global public health and peace and security scholarship and may help open discursive space for greater accountability, stronger civilian protection, and more rights-respecting, equity-centered crisis governance, even under conditions of protracted emergency. At a moment when security logics are increasingly institutionalized across health, humanitarian, and economic governance, such recognition is critical to preventing the routinization of health harm as an acceptable feature of crisis management.

## Supplementary Information

Below is the link to the electronic supplementary material.

Supplementary Material 1

## Data Availability

All primary documents analyzed in this study are publicly available and are listed in Table 1 with stable identifiers or links where available.

## References

[1] Persaud A, Bhat PS, Ventriglio A, Bhugra D. Geopolitical determinants of health. Ind Psychiatry J. 2018;27(2):308–10. 31359990 10.4103/ipj.ipj_71_18 PMC6592191

[2] Sturm T, Mercille J, Albrecht T, Cole J, Dodds K, Longhurst A. Interventions in critical health geopolitics: Borders, rights, and conspiracies in the COVID-19 pandemic. Political Geogr. 2021;91:102445. 10.1016/j.polgeo.2021.102445 PMC8580506 34785870

[3] Duong L, Sanderson HS, Phillips W, Roehrich JK, Uwalaka V. Achieving resilient supply chains: Managing temporary healthcare supply chains during a geopolitical disruption. Int J Oper Prod Manag. 2025;45(5):1090–118.

[4] Cole J, Dodds K. Unhealthy geopolitics? Bordering disease in the time of coronavirus. Geogr Res. 2021;59(2):169–81.

[5] Khorram-Manesh A, Burkle FM Jr. Civilian population victimization: A systematic review comparing humanitarian and health outcomes in conventional and hybrid warfare. Disaster Med Public Health Prep. 2023;17:e192. 10.1017/dmp.2022.96 35400358

[6] Munthe C, Fumagalli D, Malmqvist E. Sustainability principle for the ethics of healthcare resource allocation. J Med Ethics. 2021;47(2):90–7. 33154090 10.1136/medethics-2020-106644 PMC7848061

[7] Rea D, Froehle C, Masterson S, Stettler B, Fermann G, Pancioli A. Unequal but fair: Incorporating distributive justice in operational allocation models. Prod Oper Manag. 2021;30(7):2304–20.

[8] International Committee of the Red Cross. The Geneva conventions and their commentaries [Internet]. [cited 2026-01-30]. Available from: https://www.icrc.org/en/law-and-policy/geneva-conventions-and-their-commentaries.

[9] World Health Organization. Constitution of the World Health Organization. Geneva: WHO; 1948.

[10] UN Committee on Economic, Social and Cultural Rights. General comment No. 14: The right to the highest attainable standard of health (Art. 12). UN Doc. E/C.12/2000/4. 2000.

[11] World Health Organization. International Health Regulations (2005). 3rd ed. Geneva: WHO; 2016.

[12] Van Dijk TA. Critical discourse analysis. In: Tannen D, Hamilton HE, Schiffrin D, editors. The Handbook of Discourse Analysis. Hoboken (NJ): Wiley-Blackwell; 2015. pp. 466–85.

[13] Jacobs K. Discourse analysis. In: Baum S, editor. Methods in urban analysis. Singapore: Springer; 2021. pp. 151–172. 10.1007/978-981-16-1677-8_9

[14] Masic I. Public health aspects of global population health and well-being in the 21st century regarding determinants of health. Int J Prev Med. 2018;9:4. 29441181 10.4103/ijpvm.IJPVM_476_17 PMC5801579

[15] Lal A, Erondu NA, Heymann DL, Gitahi G, Yates R. Fragmented health systems in COVID-19: Rectifying the misalignment between global health security and universal health coverage. Lancet. 2021;397(10268):61–7. 33275906 10.1016/S0140-6736(20)32228-5 PMC7834479

[16] Lauriola P, Martín-Olmedo P, Leonardi GS, Bouland C, Verheij R, Dückers ML, et al. On the importance of primary and community healthcare in relation to global health and environmental threats: Lessons from the COVID-19 crisis. BMJ Glob Health. 2021;6(3):e004111. 33692145 10.1136/bmjgh-2020-004111 PMC7948151

[17] Velazquez-Meza ME, Galarde-López M, Carrillo-Quiróz B, Alpuche-Aranda CM. Antimicrobial resistance: One Health approach. Vet World. 2022;15(3):743. 35497962 10.14202/vetworld.2022.743-749 PMC9047147

[18] Walsh TR, Gales AC, Laxminarayan R, Dodd PC. Antimicrobial resistance: Addressing a global threat to humanity. PLoS Med. 2023;20:e1004264. 37399216 10.1371/journal.pmed.1004264 PMC10317217

[19] Rushton S. Security and public health. Cambridge: Polity; 2019.

[20] Holst J, van de Pas R. The biomedical securitization of global health. Global Health. 2023;19:15. 10.1186/s12992-023-00915-y. 36871029 10.1186/s12992-023-00915-y PMC9985490

[21] Ingram A. The new geopolitics of disease: between global health and global security. Geopolitics. 2005;10(3):522–45. 10.1080/14650040591003516.

[22] Nunes J. Critical security studies and global health. In: McInnes C, Lee K, Youde J, editors. The Oxford handbook of global health politics. Oxford: Oxford University Press; 2018. pp. 161–77.

[23] Akhavein D, Sheel M, Abimbola S. Health security: why is public health not enough? Glob Health Res Policy. 2025;10:1. 10.1186/s41256-024-00394-7. 39754216 10.1186/s41256-024-00394-7 PMC11697965

[24] Akhavein D, Conda EAL, Valenzuela S, et al. Governing health through security in the Philippines: a realist analysis. Health Policy Plan. 2026;41(2):262–74. 10.1093/heapol/czaf110. 41383105 10.1093/heapol/czaf110 PMC12906756

[25] Buzan B, Wæver O, de Wilde J. Security: a new framework for analysis. Boulder: Lynne Rienner; 1998.

[26] Agamben G. State of exception. Chicago: University of Chicago Press; 2005.

[27] Penz P, Drydyk J, Bose PS. Displacement by development: Ethics, rights and responsibilities; Cambridge University Press. 2011. p. 210-242..

[28] Library of Congress. Withdrawal of Germany from the league of nations. letter from konstantin von neurath [Internet]. [cited 2026-01-30]. Available from: https://www.loc.gov/item/2021667903/.

[29] Arolsen Archives. International Center on Nazi Persecution. The powerlessness of the ICRC during the Nazi period [Internet]. [cited 2026-01-30]. Available from: https://arolsen-archives.org/en/news/the-powerlessness-of-the-icrc-during-the-nazi-period/.

[30] Ingram A. The geopolitics of disease. Geogr Compass. 2009;3(6):2084–97.

[31] Koopman S, Dalby S, Megoran N, Sharp J, Kearns G, Squire R, et al. Critical geopolitics/critical geopolitics 25 years on. Political Geogr. 2021;91:102445.

[32] Cha VD. Allied decoupling in an era of US–China strategic competition. Chin J Int Polit. 2020;13(4):509–36.

[33] Wu C. Decoding US–China strategic competition: Comparative leverages and issue selection. Chin J Int Polit. 2023;16(1):31–60.

[34] Bahi R. The geopolitics of COVID-19: US–China rivalry and the imminent Kindleberger trap. Rev Econ Polit Sci. 2021;6(1):76–94.

[35] Kuzio T. Imperial nationalism as the driver behind Russia’s invasion of Ukraine. Nations Natl. 2023;29(1):30–8.

[36] Bauer Y. The Russo–Ukrainian war through a historian’s eyes. Isr J Foreign Aff. 2022;16(1):15–8.

[37] Maurer H, Whitman RG, Wright N. The EU and the invasion of Ukraine: A collective responsibility to act? Int Aff. 2023;99(1):219–38.

[38] Dov Bachmann SD, Putter D, Duczynski G. Hybrid warfare and disinformation: A Ukraine war perspective. Glob Policy. 2023;14(5):858–69.

[39] Khorram-Manesh A, Goniewicz K, Burkle FM Jr. Social and healthcare impacts of the Russian-led hybrid war in Ukraine: A conflict with unique global consequences. Disaster Med Public Health Prep. 2023;17:e432. 37476992 10.1017/dmp.2023.91

[40] Bhowmik S, Fisher J. Framing the Israel–Palestine conflict 2021: Investigation of CNN’s coverage from a peace journalism perspective. Media Cult Soc. 2023;45(5):1019–35.

[41] Mahwati T, Nanda AR. Analysis of the Palestinian–Israeli conflict in the perspective of international humanitarian law. Int Law Discourse Southeast Asia. 2022;1(1):23–42.

[42] Di Maio M, Leone Sciabolazza V. Conflict exposure and health: Evidence from the Gaza Strip. Health Econ. 2021;30(9):2287–95. 34085365 10.1002/hec.4364 PMC8453537

[43] Atique I. Discourse and controversy in the Israel–Palestine conflict: A review of the literature. 2024. Available from: https://uwindsor.scholaris.ca/items/4a3a94c3-52e5-43d7-9d39-28ce727d32d8.

[44] U.S. Department of the Treasury, Office of Foreign Assets Control (OFAC). Iran Sanctions—Sanctions programs and country information [Internet]. [cited 2026-01-30]. Available from: https://ofac.treasury.gov/sanctions-programs-and-country-information/iran-sanctions.

[45] Iran Human Rights (IHRNGO). One year protest report: at least 551 killed and 22 suspicious deaths [Internet]. 2023 Sep 15 [cited 2026-01-30]. Available from: https://www.iranhr.net/en/print/5/6200/.

[46] Kaye DD, Wehrey FM. A nuclear Iran: The reactions of neighbours. Survival. 2007;49(2):111–28.

[47] Associated Press. Activists say Iran’s crackdown has killed at least 6,221 people, as the country’s currency plunges [Internet]. 2026 Jan [cited 2026-01-30]. Available from: https://apnews.com/article/aeaeb26493d25d86d5169f8ae455e405.

[48] Congress.gov. Venezuela: Overview of the US sanctions policy [Internet]. [cited 2026-01-30]. Available from: https://www.congress.gov/crs-product/IF10715.

[49] US department of treasury. Office of foreign assets control. Venezuela related sanctions [Internet]. [cited 2026-01-30]. Available from: https://ofac.treasury.gov/sanctions-programs-and-country-information/venezuela-related-sanctions.

[50] Sokolshchik LM, Sokolshchik YS, Teremetskiy KS, US Sanctions Policy Towards Latin America. Cases of Official Narratives on Cuba, Venezuela, and Nicaragua. Vestnik Volgogradskogo gosudarstvennogo universiteta Ser 4 Istoriya Regionovedenie Mezhdunarodnye otnosheniya. 2024;29(1):214–24.

[51] Paulus T, Woods M, Atkins DP, Macklin R. The discourse of QDAS: Reporting practices of ATLAS.ti and NVivo users with implications for best practices. Int J Soc Res Methodol. 2017;20(1):35–47.

[52] U.S. Food and Drug Administration. Executive order 14017 on America’s supply chains [Internet]. [cited 2026-01-30]. Available from: https://www.fda.gov/about-fda/reports/executive-order-14017-americas-supply-chains.

[53] The American Presidency Project (UC Santa Barbara). Executive order 14336—ensuring American pharmaceutical supply chain resilience by filling the strategic active pharmaceutical ingredients reserve [Internet]. 2025 Aug 13 [cited 2026-01-30]. Available from: https://www.presidency.ucsb.edu/documents/executive-order-14336-ensuring-american-pharmaceutical-supply-chain-resilience-filling-the.

[54] U.S. Department of Defense (via DAU). Report—HASC, critical supply chain [Internet]. [cited 2026-01-30]. Available from: https://www.dau.edu/sites/default/files/Migrated/CopDocuments/Report%20-%20HASC%2C%20Critical%20Supply%20Chain.pdf.

[55] The White House. Ensuring American pharmaceutical supply chain resilience by filling the strategic active pharmaceutical ingredients reserve [Internet]. 2025 Aug 13 [cited 2026-01-30]. Available from: https://www.whitehouse.gov/presidential-actions/2025/08/ensuring-american-pharmaceutical-supply-chain-resilience-by-filling-the-strategic-active-pharmaceutical-ingredients-reserve/.

[56] The Print. Full text of Vladimir Putin’s speech announcing ‘special military operation’ in Ukraine [Internet]. 2022 Feb 24 [cited 2026-01-30]. Available from: https://theprint.in/world/full-text-of-vladimir-putins-speech-announcing-special-military-operation-in-ukraine/845714/.

[57] World Health Organization (Europe). Three years of war: rising demand for mental health support, trauma care and rehabilitation [Internet]. 2025 Feb 24 [cited 2026-01-30]. Available from: https://www.who.int/europe/news/item/24-02-2025-three-years-of-war-rising-demand-for-mental-health-support-trauma-care-and-rehabilitation.

[58] WHO. Ukraine: WHO Health Emergency Appeal 2025 (January 2025) [Internet]. 2025 Jan [cited 2026-01-30]. Available from: https://reliefweb.int/report/ukraine/ukraine-who-health-emergency-appeal-2025-january-2025.

[59] United Nations Office for the Coordination of Humanitarian Affairs (UN OCHA). Flash appeal: occupied Palestinian territory, April–December 2024 [Internet]. 2024 Apr 17 [cited 2026-01-30]. Available from: https://www.unocha.org/publications/report/occupied-palestinian-territory/flash-appeal-occupied-palestinian-territory-april-december-2024-april-2024.

[60] United Nations Security Council. Resolution 2712 (2023) / adopted by the Security Council at its 9479th meeting, 15 November 2023 [Internet]. 2023 [cited 2026-01-30]. Available from: https://digitallibrary.un.org/record/4027698.

[61] United Nations. Adopting Resolution 2712 (2023), Security council calls for ‘urgent and extended’ humanitarian pauses in Gaza, immediate release of hostages (SC/15496) [Internet]. 2023 Nov 15 [cited 2026-01-30]. Available from: https://press.un.org/en/2023/sc15496.doc.htm.

[62] World Health Organization (EMRO). April 2024–December 2024 WHO operational response plan: occupied Palestinian territory [Internet]. 2024 [cited 2026-01-30]. Available from: https://www.emro.who.int/images/stories/palestine/WHO_WHO_oPt_Operational_Plan_April_2024.pdf.

[63] United Nations Office for the Coordination of Humanitarian Affairs. Flash appeal: occupied Palestinian territory, April–December 2024 [Internet]. 2024 [cited 2026-01-30]. Available from: https://www.unocha.org/publications/report/occupied-palestinian-territory/flash-appeal-occupied-palestinian-territory-april-december-2024-april-2024.

[64] Usmany J, Barten DG, Goniewicz K, et al. Attacks on healthcare in conflict-affected countries: a comparison of temporal trends in ongoing conflicts in Lebanon, Myanmar, occupied Palestinian territory, Sudan and Ukraine using WHO SSA and SHCC data, 2018–2024. Popul Health Metrics. 2026;24:5. 10.1186/s12963-025-00442-5. 10.1186/s12963-025-00442-5 PMC12822187 41392142

[65] Associated Press / PBS NewsHour. Trump cancels meetings with Iranian officials and tells protesters “help is on its way” [Internet]. 2026 Jan [cited 2026-01-30]. Available from: https://www.pbs.org/newshour/world/trump-cancel-meetings-with-iranian-officials-and-tells-protesters-help-is-on-its-way.

[66] Reuters/Yahoo News. Trump encourages Iran protesters, says “help is on its way” [Internet]. 2026 Jan [cited 2026-01-30]. Available from: https://www.yahoo.com/news/articles/trump-encourages-iran-protesters-says-155826876.html.

[67] U.S. Department of the Treasury, OFAC. Guidance related to the provision of humanitarian assistance and support to the venezuelan people [Internet]. 2019 [cited 2026-01-30]. Available from: https://ofac.treasury.gov/media/26781/download?inline=.

[68] U.S. Department of the Treasury. Treasury sanctions maduro regime officials tied to repression [Internet]. 2024 (or posted date on page) [cited 2026-01-30]. Available from: https://home.treasury.gov/news/press-releases/jy0218.

[69] U.S. Department of State. Venezuela-Related sanctions [Internet]. [cited 2026-01-30]. Available from: https://www.state.gov/venezuela-related-sanctions/.

[70] U.S. Department of State (archived). United States opposes maduro’s illegitimate attempt to seize power in Venezuela [Internet]. 2021 Jan 5 [cited 2026-01-30]. Available from: https://state.gov/united-states-opposes-maduros-illegitimate-attempt-to-seize-power-in-venezuela/.

[71] Rodríguez F. The human consequences of economic sanctions. J Econ Stud. 2024;51(4):942–63.

[72] Tarigan J, Delia M, Hatane SE. Impact of the Russia–Ukraine War: evidence from G20 countries. Stud Econ Finance. 2025;42(1):135–53.

[73] United Nations Office on Drugs and Crime (UNODC). Key issues: defining terrorism. United Nations office on drugs and crime. 2023. [cited 2026-01-30]. Available from: https://www.unodc.org/e4j/en/terrorism/module-4/key-issues/defining-terrorism.html.

[74] Schmidt, A. Defining terrorism. International Centre for Counter-Terrorism. 2023. [cited 2026-01-30]. Available from: https://icct.nl/publication/defining-terrorism.

[75] Shamsi H. ACLU. How NSPM-7 seeks to use domestic terrorism to target nonprofits and activists. 2025. [cited 2026-01-30]. Available from: https://www.aclu.org/news/national-security/how-nspm-7-seeks-to-use-domestic-terrorism-to-target-nonprofits-and-activists.

[76] Haas, M. Labeling dissent as terrorism: New U.S. domestic terrorism priorities raise constitutional alarms. The conversation. 2025. [cited 2026-01-30]. Available from: https://theconversation.com/labeling-dissent-as-terrorism-new-us-domestic-terrorism-priorities-raise-constitutional-alarms-269161.

[77] Grinko M, Qalandar Randall D, Wulf V. Nationalizing the internet to break a protest movement: Internet shutdown and counter-appropriation in iran of late 2019. Proceedings of the ACM on human-computer interaction. 2022;6(CSCW2): 1-21.

[78] O’Neil C. Iran’s digital fortress: the rise of the national information network. Iran strategy brief, American foreign policy council. 2025; 3-4. https://www.afpc.org/uploads/documents/Iran_Strategy_Brief_No._16_-_August_2025.pdf https://www.afpc.org/uploads/documents/Iran_Strategy_Brief_No._16_-_August_2025.pdf

[79] Khatib R, McKee M, Yusuf S. Counting the dead in Gaza: difficult but essential. Lancet. 2024;404(10449):237–8. 38976995 10.1016/S0140-6736(24)01169-3

[80] Hier SP, Greenberg JL. Constructing a discursive crisis: Risk, problematization and illegal Chinese in Canada. Ethnic racial Stud. 2002;25(3):490–513.

[81] Trelles M, Stewart BT, Kushner AL. Attacks on civilians and hospitals must stop. The lancet global health. 2016;4(5): e298-e299. 10.1016/S2214-109X(16)00070-X 27012677

[82] Khorram-Manesh A, Burkle FM Jr. The unprecedented purposeful targeting of health systems and hospitals in wars and conflicts: an immediate call for global intervention. *Journal of Health and Human Experience*, 2024;1103. Available from: https://jhhe.sempervifoundation.org/pdfs/JHHE%20V10n1%20Spring2024%20LowResPages_3.pdf#page=113 .

[83] Bliuc AM, Chidley A. From cooperation to conflict: The role of collective narratives in shaping group behaviour. Soc Pers Psychol Compass. 2022;16(7):e12670.

[84] De Vries CE. How foundational narratives shape European Union politics. JCMS: J Common Market Stud. 2023;61(4):867–81.

[85] Newsom VA, Lengel L, Birzescu A, Vukasovich C. Strategic narratives in political and crisis communication: responses to COVID-19. Frontiers in communication. 2022;7: 1000359.

[86] Brennan R, Sheraz M. The international community is failing to protect healthcare in armed conflict. BMJ. 2024;387:q2474. 10.1136/bmj.q2474 PMC11555554 39532512

[87] Bruneau E, Kteily N. The enemy as animal: Symmetric dehumanization during asymmetric warfare. PLoS ONE. 2017;12(7):e0181422. 28746412 10.1371/journal.pone.0181422 PMC5528981

[88] Kteily N, Hodson G, Bruneau E. They see us as less than human: Metadehumanization predicts intergroup conflict via reciprocal dehumanization. J Personal Soc Psychol. 2016;110(3):343–70. 10.1037/pspa0000044 26963763

[89] Rai TS, Valdesolo P, Graham J. Dehumanization increases instrumental violence, but not moral violence. Proc Natl Acad Sci U S A. 2017;114(32):8511–8516. 10.1073/pnas.1705238114 PMC5559031 28739935

[90] Goniewicz K, Khorram-Manesh A, Burkle JR. FM. Beyond boundaries: Addressing climate change, violence, and public health. Prehosp Disaster Med. 2023;38(5):551–4. 37650224 10.1017/S1049023X23006271

[91] Khorram-Manesh A. Global transition, global risks, and the UN’s sustainable development goals–A call for peace, justice, and political stability. Global Transitions. 2023;5:90–7.

[92] Bužinkić E, Foley J, Kerr E. Crisis, Governance, (De) Mobilisation and New Inequalities: The Legacy of COVID-19. Crit Sociol. 2025;51(1):105–14.

[93] Khorram-Manesh A, Burkle FM, Goniewicz K, Robinson Y. Estimating the number of civilian casualties in modern armed conflicts–a systematic review. Front public health. 2021;9:765261. 34778192 10.3389/fpubh.2021.765261 PMC8581199

