# Supplementary material for: Security governance and health inequity in contemporary crises: a critical discourse analysis of securitization, exception-making, and responsibility displacement
Source: Global Health. 2026 Jun 23;22:55. doi: 10.1186/s12992-026-01226-8 (PMC13309965; doi:10.1186/s12992-026-01226-8)
Supplement: Supplementary file 1 — Supplementary Material 1 [file 12992_2026_1226_MOESM1_ESM.docx]

**Supplementary materials**

**Supplementary Tables 1-4**

**Supplementary Table 1. NVivo coding framework for the critical discourse analysis**

This supplementary table summarizes the coding framework used to organize and interpret the primary corpus in NVivo. The framework was developed through a combination of inductive open coding and theory-informed focused coding. It was used to identify how institutional and policy discourse represented health-relevant governance during security-framed crises. The aim was not to quantify the frequency of terms, but to examine how language constructed crisis priorities, legitimized exceptional measures, distributed responsibility, and shaped the perceived boundaries of humanitarian access, civilian protection, and health equity.

| Parent code | Working definition | Textual indicators / search prompts | Analytical question | Example coding logic |
| --- | --- | --- | --- | --- |
| 1. Securitization | Language that represents health-relevant domains as threats, strategic vulnerabilities, or security assets requiring urgent protection, control, or exceptional governance. | “security,” “national security,” “threat,” “risk,” “resilience,” “strategic,” “vulnerability,” “dependency,” “sovereignty,” “adversary,” “border,” “stability,” “critical infrastructure.” | How is health, humanitarian access, medical supply, or civilian protection reframed as a security matter? | A passage linking pharmaceutical supply chains to “national security” would be coded as securitization because health goods are represented as strategic assets rather than primarily as public goods. |
| 1.1 Health as strategic asset | Health systems, medical goods, hospitals, or supply chains are framed as assets to be protected for strategic or national interests. | “medical product security,” “critical supply chains,” “strategic reserve,” “resilience,” “self-sufficiency.” | Does the text treat health infrastructure as part of national or geopolitical competition? | Used especially in US–China supply-chain discourse. |
| 1.2 Threat framing | A crisis actor, population, supply route, border, protest, or institution is described through threat language. | “enemy,” “terrorist,” “destabilizing,” “foreign interference,” “hostile,” “adversary,” “threat to stability.” | Who or what is constructed as dangerous? | Used where protest, migration, sanctions targets, or rival states are framed as threats. |
| 1.3 Strategic dependency | Interdependence is framed as vulnerability or exposure to hostile actors. | “dependency,” “overreliance,” “foreign concentration,” “supply-chain risk,” “insulation.” | How does the text transform interdependence into a security problem? | Used in pharmaceutical, technology, and medical-supply discourse. |
| 2. Exception-making | Language that authorizes or normalizes departures from ordinary protection norms through claims of necessity, emergency, temporariness, proportionality, or feasibility. | “temporary,” “urgent,” “necessary,” “proportionate,” “emergency,” “exception,” “pause,” “corridor,” “authorization,” “license,” “exemption,” “special measure.” | How does the text justify limited, conditional, or temporary protection? | A passage referring to “humanitarian pauses” instead of continuous protection would be coded as exception-making. |
| 2.1 Temporary protection | Protection is framed as time-limited, conditional, or episodic. | “pause,” “temporary,” “limited,” “short-term,” “extended pause,” “window,” “corridor.” | Does protection appear as an exception rather than a standing obligation? | Used in Gaza humanitarian access discourse. |
| 2.2 Humanitarian exemption / licensing | Humanitarian access is framed as permitted through special authorization within a broader restrictive regime. | “license,” “general license,” “exemption,” “authorized transactions,” “permitted exports,” “humanitarian channel.” | Is humanitarian activity treated as a special exception rather than a baseline obligation? | Used in Iran and Venezuela sanctions guidance. |
| 2.3 Emergency necessity | Measures are justified as unavoidable because of crisis, threat, war, or emergency. | “necessity,” “emergency,” “urgent need,” “exceptional circumstances,” “security imperative.” | What is made acceptable by invoking emergency? | Used across conflict, sanctions, and supply-chain texts. |
| 3. Responsibility displacement | Language that diffuses, obscures, or relocates agency and accountability for health harms by presenting them as indirect, technical, logistical, market-based, or politically contested. | “operational constraints,” “access challenges,” “market conditions,” “logistical barriers,” “conflict environment,” “unintended consequences,” passive voice, unclear actor attribution. | How are harms separated from identifiable decisions, actors, or policies? | A passage saying aid failed because of the “operating environment” may be coded as responsibility displacement if it obscures who created or maintained that environment. |
| 3.1 Passive agency | Harm is described without clearly naming the actor responsible. | “facilities were damaged,” “access was restricted,” “services were disrupted,” “people were displaced.” | Is agency grammatically or politically obscured? | Used where attacks or restrictions are described without naming responsible actors. |
| 3.2 Technical/logistical framing | Political constraints are described as technical or operational problems. | “logistics,” “coordination,” “implementation challenges,” “delivery barriers,” “administrative delays.” | Does the text depoliticize a governance choice by presenting it as technical? | Used in humanitarian and sanctions discourse. |
| 3.3 Market/intermediary displacement | Responsibility is shifted to banks, suppliers, insurers, or market actors. | “de-risking,” “compliance risk,” “payment friction,” “supplier reluctance,” “banking channels.” | Are predictable policy effects treated as market behavior? | Used in sanctions-related humanitarian access. |
| 3.4 Contested mortality and attribution | Death counts, injury figures, or responsibility claims are presented as disputed, uncertain, or politically contested. | “disputed death toll,” “activists say,” “officials deny,” “unverified,” “attributed to,” “conflicting reports.” | How does uncertainty over counting or attribution shape accountability? | Used in Iran and Gaza-related mortality discussions. |
| 4. Counter-framing / rights-based contestation | Language that resists security-dominant framing by emphasizing rights, equity, humanitarian principles, civilian protection, solidarity, or health as a public good. | “right to health,” “civilian protection,” “humanitarian obligation,” “public good,” “equity,” “non-discrimination,” “universal access,” “solidarity.” | Where does the text contest, qualify, or limit securitized governance? | WHO or UN passages emphasizing universal access may be coded here, especially where later narrowed by security conditions. |
| 4.1 Health as public good | Health goods, technologies, services, or knowledge are framed as shared or universal goods. | “public good,” “solidarity,” “equitable access,” “shared resources,” “global cooperation.” | Does the text offer an alternative to national-security framing? | Used in WHO C-TAP and global health governance texts. |
| 4.2 Civilian protection norms | The text invokes IHL, humanitarian principles, civilian protection, or non-derogable obligations. | “civilian protection,” “international humanitarian law,” “medical neutrality,” “protection of healthcare,” “humanitarian principles.” | How are protection norms asserted, and are they later narrowed? | Used in UN, WHO, OCHA, and ICRC-relevant discourse. |
| 4.3 Equity and access | The text foregrounds unequal access, vulnerability, or distributional harm. | “equity,” “access,” “vulnerable populations,” “disproportionate impact,” “health inequity.” | How does the text make inequity visible? | Used across health emergency appeals and humanitarian reports. |

**Example coding rule**

A single passage could receive more than one code. For example:

“Humanitarian assistance is authorized through specific licenses, subject to compliance requirements.”

This could be coded as:

Exception-making → Humanitarian exemption/licensing
because humanitarian activity is framed as permitted through special authorization.

Responsibility displacement → Market/intermediary displacement
if the surrounding text emphasizes compliance risk, banking caution, or supplier hesitation.

Counter-framing → Equity and access
if the passage also refers to protecting civilian access to medicine or food.

This reflects the CDA assumption that meanings overlap and that the same passage can simultaneously authorize protection, constrain it, and shift responsibility for its failure.

Formulärets överkant

Formulärets nederkant

**Supplementary Table 2. Trustworthiness and analytical safeguards in the critical discourse analysis**

| Rigour domain | What was done (procedures) | What it addresses (why it matters) | Where evidenced in manuscript / supplement |
| --- | --- | --- | --- |
| Audit trail | Maintained NVivo memos documenting analytic decisions, evolving interpretations, and code revisions across iterative coding cycles. | Makes interpretive steps transparent and traceable; reduces “black box” critique of CDA. | Methods section 2.7; Supplementary Table 3 |
| Iterative code development | Progressed from open coding (frames, metaphors, modality, agency patterns) to focused coding aligned with securitisation, exception-making, and responsibility displacement; refined codebook iteratively. | Demonstrates systematic movement from text-level features to theoretically informed mechanisms; reduces post-hoc “fit.” | Methods sections 2.3 and 2.5; Supplementary Table 1 |
| Cross-source triangulation | Compared discursive patterns across institutional issuers (state communications, WHO, UN bodies, sanctions guidance, NGO reporting) and used secondary scholarship as interpretive context (not coded). | Limits over-reliance on one institutional voice; strengthens plausibility of cross-case mechanisms. | Methods sections 2.3 and 2.7; Table 1 |
| Cross-case comparison | Conducted structured comparisons across the five case contexts to identify recurring mechanisms and context-specific articulation (lexical choices, qualifiers, responsibility frames). | Guards against overgeneralisation from a single case; clarifies what travels across contexts and what is contextual. | Results sections 3.1–3.6 |
| Excerpt selection strategy | Selected 1–4 illustrative excerpts per case based on recurrence (frequent phrases/frames), institutional salience (authoritative issuers), and representativeness of the mechanism; kept excerpts short and linked to codes. | Reduces “cherry-picking” concerns; makes evidence-to-claim linkage explicit. | Methods section 2.7; Results case subsections; Supplementary Table 4 |
| Handling contested mortality and attribution | Treated counting, naming, attribution, and competing death toll claims as part of the discursive field; analysed how uncertainty/contestation can displace responsibility. | Prevents importing unstable baselines as “facts” when the politics of verification is itself governance-relevant; strengthens validity in closed contexts. | Methods section 2.7; Iran results subsection; Supplementary Table 4 |
| Negative/deviant cases | Recorded instances where institutional texts invoked rights/equity language that partially resisted securitisation; analysed how these counter-frames were narrowed or operationally conditioned. | Shows analytic openness; prevents forcing all evidence into one mechanism; increases credibility. | Discussion paragraph on rights-based and humanitarian counter-framing; Supplementary Table 1 |
| Reflexivity | Maintained reflexive notes on interpretive choices, positionality, and potential analytic blind spots (e.g., institutional vantage vs lived experience). | Addresses CDA critique that interpretation is arbitrary; clarifies limits of inference. | MMethods sections 2.3 and 2.7ethods (Trustworthiness and rigour); optional S2 File: reflexive note excerpt. |

**Supplementary Table 3. Example audit trail for CDA coding and interpretation**

| Stage | Analytic activity | Decision or observation | Rationale | Action taken |
| --- | --- | --- | --- | --- |
| Corpus construction | Reviewed institutional documents across five cases | Included WHO, UN, OFAC, state, and NGO/human-rights documents; excluded general commentary | Focus was on authoritative institutional discourse | Finalized primary corpus and classified documents by actor type |
| Open coding | First-cycle coding in NVivo | Initial codes included security, risk, resilience, temporary protection, humanitarian access, operational constraints, and contested mortality | These terms were recurrent and relevant to the research question | Created initial NVivo code list |
| Memo writing | Wrote analytic memos during coding | Noted that “humanitarian pauses” functioned differently from rights-based protection language | The wording suggested bounded and temporary protection | Developed the focused code “exception-making” |
| Code refinement | Reviewed overlapping codes | Some passages about sanctions exemptions also contained compliance-risk language | The same text could authorize humanitarian access while creating administrative friction | Allowed multiple codes for one passage |
| Cross-case comparison | Compared coded passages across cases | State texts used threat and legitimacy language; WHO/UN texts used operational language; sanctions authorities used licensing/compliance language | Actor types used different genres of discourse | Added actor-type comparison to Methods and Results |
| Contested mortality | Reviewed Iran and Gaza-related mortality passages | Mortality figures were often disputed or attributed differently by different actors | Contestation itself was part of the governance discourse | Treated counting, attribution, and naming as part of responsibility displacement |
| Co-author review | K.G. reviewed selected coded documents and memos | Some code applications needed clearer distinction between securitization and exception-making | Improved conceptual consistency | Revised code definitions and coding framework |
| Final interpretation | Synthesized findings | Three recurring mechanisms and one counter-framing category were synthesized | These mechanisms recurred across cases but operated differently by actor and context | Finalized Results structure and illustrative excerpts |

**Supplementary Table 4. Examples of coded passages**

These examples illustrate how short passages were linked to codes in NVivo. They are illustrative rather than exhaustive, and codes were not mutually exclusive.

| Source type | Example coded passage | Codes assigned | Coding rationale | Analytic interpretation |
| --- | --- | --- | --- | --- |
| UN Security Council / Gaza | “urgent and extended humanitarian pauses and corridors” | Exception-making; temporary protection | Protection is framed as temporary and conditional | Humanitarian protection becomes operationally possible but bounded |
| Sanctions guidance | “authorized transactions for agricultural commodities, medicine, and medical devices” | Exception-making; humanitarian exemption/licensing | Humanitarian access is permitted through authorization within a restrictive regime | Access appears possible in principle but dependent on compliance architecture |
| WHO emergency reporting | “access constraints and operational barriers” | Responsibility displacement; operational/logistical framing | Harm is described through technical or logistical language | Health disruption is visible, but political responsibility may be backgrounded |
| FDA/supply-chain discourse | “secure, robust, and resilient medical product supply chains are essential for health and national security” | Securitization; health as strategic asset | Medical products are explicitly linked to national security | Health goods are framed as strategic assets rather than only as public goods |
| Human-rights mortality reporting | “verified the killing of 551 protesters including 68 children” | Contested mortality and attribution; accountability documentation | Mortality is documented in a politically contested setting | Counting and attribution become part of accountability struggle |
